# POINT-OF-CARE TESTING FOR THE FRAILTY PHENOTYPE PREDICTS POSTSURGICAL OUTCOMES

**DOI:** 10.1093/geroni/igac059.1568

**Published:** 2022-12-20

**Authors:** Mamoun Mardini, Christopher Kaufmann

## Abstract

Frailty is a cornerstone in geriatric medicine and is known to be strongly associated with poor postsurgical outcomes. Fried and colleagues defined frailty as a syndrome with five interlinked components, including weakness, low energy, slow walking speed, decreased physical activity, and unintentional weight loss. Consequently, frailty could serve as a useful measure for the purposes of pre-probability risk assessment for clinical outcomes after a surgery. Electronic health records (EHR) are a rich source of information, and when harnessed appropriately, could provide a valid presurgical frailty screening metric and be used towards anticipating adverse postsurgical outcomes. The growing adoption of EHR in clinical settings can help in identifying patients in need of enhanced intervention to promote recovery following a surgery. However, to date, few studies have fully characterized how individual frailty components, as assessed in the clinic, could be a predictor of postsurgical outcomes. This symposium will examine how presence of frailty and its individual components predict three postsurgical clinical outcomes: length of stay, 30-day mortality, and discharge disposition. We will present data from a unique dataset of approximately 14,000 patients who underwent frailty assessment at the University of Florida-Health (UFHealth) presurgical clinic as part of their standard clinical evaluation. Our presentations will examine each frailty syndromic components and overall frailty as predictors of the outcomes of interest. Findings will demonstrate the utility of electronic health records data for the allocation of at-risk patients for poor postsurgical outcomes. Funding: UF Claude D. Pepper Older Americans Independence Center P30AG028740 and R01 AG055337.

